# Solute transporters and malignancy: establishing the role of uptake transporters in breast cancer and breast cancer metastasis

**DOI:** 10.1007/s10555-020-09879-6

**Published:** 2020-05-09

**Authors:** Rachel Sutherland, Annette Meeson, Simon Lowes

**Affiliations:** 1 Biosciences Institute, Newcastle University, International Centre for Life, Central Parkway, Newcastle Upon Tyne, UK; 2 Translational and Clinical Research Institute, Medical School, Newcastle University, Framlington Place, Newcastle Upon Tyne, UK; 3 Breast Screening and Assessment Unit, Queen Elizabeth Hospital, Gateshead Health NHS Foundation Trust, Gateshead, Sheriff Hill, UK

**Keywords:** Breast cancer, SLCO, SLC22, Drug transport, Chemotherapeutic drugs, Oestrogen transport

## Abstract

The solute carrier (SLC) superfamily encompasses a large variety of membrane-bound transporters required to transport a diverse array of substrates over biological membranes. Physiologically, they are essential for nutrient uptake, ion transport and waste removal. However, accumulating evidence suggest that up- and/or downregulation of SLCs may play a pivotal role in the pathogenesis of human malignancy. Endogenous substrates of SLCs include oestrogen and its conjugates, the handling of which may be of importance in hormone-dependent cancers. The SLCs play a significant role in the handling of therapeutic agents including anticancer drugs. Differential SLC expression in cancers may, therefore, impact on the efficacy of treatments. However, there is also a small body of evidence to suggest the dysregulated expression of some of these transporters may be linked to cancer metastasis. This review draws on the current knowledge of the roles of SLC transporters in human cancers in order to highlight the potential significance of these solute carriers in breast cancer pathogenesis and treatment.

**Figure:**
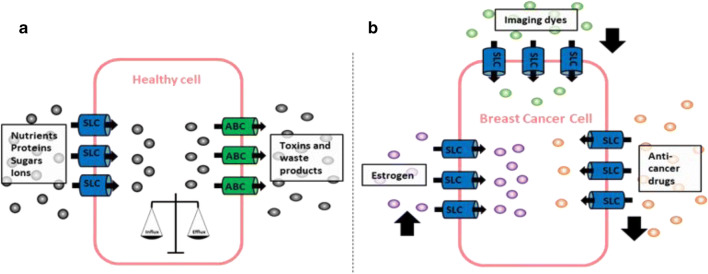
Graphical abstract

Graphical abstract

## Introduction

A spectrum of mechanisms underpin the pathogenesis of breast cancer (BC), and characterizing these can not only help us understand how BCs develop and progress but also provide potential targets for treatment. Membrane transporters (both efflux and influx) significantly influence the progression of BC but less is known about their role compared with that in other tissues.

Most studies to date have focused on the role of ABC (ATP-binding cassette) transporters in BC. These have shown that upregulation of several ABC transporters (ABCB1, ABCG2 and ABCC1) in response to chemotherapeutic agents can promote development of drug resistance [1]. However, focus has increasingly shifted to other membrane transporters that contribute to BC progression, including those that mediate substrate uptake, including SLC families that are key candidates due to their ability to transport a wide range of organic anions/cations and zwitterions [2]. Substrate specificities and differences in expression profiles of these SLC transporters between normal and cancer cells may reflect differing metabolic demands and could give clues to cell behaviour, including differences in drug sensitivities, cell proliferation and efficacy of chemotherapeutics [3].

Currently, there is relatively little information on the role of SLC transporters in BC. This review aims to draw together this limited data, together with information on other cancer types, in order to build a picture of their likely significance in BC, and to guide further research to exploit their potential as prognostic indicators and therapeutic targets.

### SLC transporters physiological expression and substrates

The SLC superfamily is the second largest family of membrane proteins. They are classified into 52 families containing 395 transporters which together are ubiquitously expressed throughout the body particularly within epithelial tissues. Two of these families will be the focus of this review, the SLCO family comprising organic anion–transporting peptides (OATP) and the SLC22A family comprising organic anionic (OAT), organic cationic (OCT) and organic carnitine/zwitterion (OCTN) transporters. They play an essential role in the maintenance of homeostasis through the uptake of solutes that do not freely diffuse across biological membranes, and are predominantly facultative transporters requiring an electrochemical gradient to drive substrate movement into cells or secondary-active transporters relying on ion gradients created by ion extrusion [4] [5].

### SLCO transporters

Eleven OATPs have been identified in humans that transport a plethora of substrates mainly organic anions and endo- and xenobiotics as outlined in Table 1.

**Table 1:** The human organic anion transporters: Gene name, corresponding transport protein, tissue distribution and main substrates [5–8]

Gene	Protein	Tissue expression	Substrates
*SLCO1A2*	OATP1A2	Breast, brain, kidney, intestine, liver, eye, lung, testis	Bile salts
			Organic anion and cations
			Bilirubin
			Steroid hormone metabolites
*SLCO1B1*	OATP1B1	Breast**,** liver, kidney	Bile salts
			Organic anions
			Bilirubin
			Steroid hormone metabolites
			Thyroid hormones
			Inflammatory mediators
*SLCO1B3*	OATP1B3	Breast, liver, kidney	Bile salts
			Organic anions
			Bilirubin
			Steroid hormones
			Thyroid hormones
			Inflammatory mediators
*SLCO1C1*	OATP1C1	Brain, testis	Thyroid hormones
			Steroid hormones
*SLCO2A1*	OATP2A1	Ubiquitous (including breast)	Prostaglandins
			Inflammatory mediators
*SLCO2B1*	OATP2b1	Liver, placenta, intestine, breast, eye, mammary gland, skin, heart, skeletal muscle, brain	E-3-S
			DHEA-S
			Steroid hormones
			Thyroid hormones
			Inflammatory mediators
*SLCO3A1*	OATP3A1	SLCO3A1_v1: testis, heart, brain, breast, ovary, lung, spleen, thyroid gland SLCO3A1_v2: testis, brain	E-3-S
			Prostaglandins
			Steroid hormones
			Thyroid hormones
			Inflammatory mediators
*SLCO4A1*	OATP4A1	Ubiquitous (including breast)	Taurocholate
			Thyroid hormones
			Prostaglandin
			Bile salts
			Steroid hormones
*SLCO4C1*	OATP4C1	Breast, kidney, liver	Bile salts
			Digoxin
			Thyroid hormones
			Methotrexate
			Steroid hormones
*SLCO5A1*	OATP5A1	Breast, foetal brain, prostate, skeletal muscle, thymus	Non-identified
*SLCO6A1*	OATP6A1	Testis, spleen, brain, foetal brain, placenta	Non-identified

### SLC22 transporters

The human SLC22 genes encode 23 transport proteins of which 13 have been well characterised. Within each subgroup (OCT, OAT and OCTN) the transporters share overlapping substrate specificities and are distributed throughout similar tissues as outlined in Table 2 [13].

**Table 2:** The human organic anionic and cationic transporters: Gene name, corresponding transport protein, tissue distribution and main substrates [9–12]

Gene	Protein	Tissue expression	Substrates
*SLC22A1*	OCT1	Liver, intestine, kidney, lung, skeletal muscle, brain, adipose tissue, immune cells	Organic cations
			Oxiplatin
			Cisplatin
			Carboplatin
*SLC22A2*	OCT2	Kidney, small intestine, lung, placenta, thymus, brain, inner ear	Organic cations
			Oxaliplatin
			Cisplatin
*SLC22A3*	OCT3	Heart, skeletal muscle, brain, small intestine, liver, lung, kidney, bladder, breast, blood vessels, placenta	Organic cations
			Oxaliplatin
*SLC22A4*	OCTN1	Kidney, intestine, spleen, heart, skeletal muscle, brain, breast, thymus, prostate, airways, testis, eye, immune cells, bone marrow, pancreas, placenta, lung	Zwitterions
			Organic cations
			l-Carnitine
			Oxaliplatin
*SLC22A5*	OCTN2	Skeletal muscle, kidney, breast, prostate, lung, pancreas, heart, small intestine, thyroid gland, liver, placenta and spinal cord	Zwitterions
			Organic cations
			l-Carnitine
*SLC22A6*	OAT1	Kidney, placenta, brain, eyes, smooth muscle	Small xenobiotics
			Prostaglandin
			Urate
			Antivirals
			Methotrexate
*SLC22A7*	OAT2	Liver, kidney, lung, brain, small intestine, eye, heart	Antivirals
			cGMP
			Prostaglandin
			Tetracycline
			Estrone-3-sulfate
*SLC22A8*	OAT3	Liver, kidney, brain, skeletal muscle, retina, testes	
			Small xenobiotic
			Conjugated steroids
			Carnitine
			Prostaglandin
			Vitamins
			Estrone-3-sulfate
			Estradiol
			Methotrexate
			Tetracycline
*SLC22A9*	OAT7	Liver	Oestrogen-3-sulfate
*SLC22A10*	OAT5	Liver	Unknown
*SLC22A11*	OAT4	Kidney, placenta, brain	Estrone-3-sulfate
			Prostaglandin
			Urate
			Tetracycline
			Methotrexate
*SLC22A12*	URAT1	Kidney, smooth muscle	Urate
*SLC22A13*	OAT10	Kidney, colon, small intestine, brain, heart	Urate
			Organic anions
			Nicotinate
*SLC22A16*	OCT6	Testis, bone marrow, kidney	l-Carnitine
			Non-charged compounds
*SLC22A20*	OCT6	Olfactory mucosa, testes	Estrone-3-sulfate

### SLC transporters and cancer

SLC transporters are expressed in many cancers and can be differentially expressed between malignant and non-malignant tissues. This has led to an interest in the roles these transporters play in cancer progression.

### OATPs

*SLCO1A2 (OATP1A2)* is present in numerous tissues and is recognised as a prostaglandin transporter. Its other substrates are steroid hormones/conjugates; estradiol-17β-glucuronide, estrone-3-sulfate (E-3-S), dehydroepiandrosterone sulfate (DHEA-S) and anticancer drugs; imatinib, methotrexate, paclitaxel, doxorubicin and docetaxel [14]. Physiologically, SLCO1A2 is expressed most highly in the brain and, to a lesser extent, in several other tissues (Table 1). In cancer, tissue distribution includes breast as well as, gliomas, colon polyps, bone, prostate, pancreatic and head and neck cancers [14–21]. Although differential expression of OATP1A2 has been found in cancer cells compared with healthy tissue, the pattern varies between cell types; in head and neck squamous cell carcinoma its expression is significantly increased compared with healthy adjacent tissue but expression appears to be lower in colonic tumours than healthy colon [16, 21]. In the prostate, SLCO1A2 may play a role in the growth of prostate cancer cells through DHEA-S uptake suggesting that inhibition of this SLCO1A2 could help to attenuate tumour cell growth [15].

SLCO1A2 protein expression has been detected in the cell membrane and cytoplasm of breast carcinoma cells but not in cells of adjacent healthy tissues. High expression was also observed in BC cell lines ZR-75-1 and T-47D [14]. A significant correlation between the expression of this transporter and the pregnane X receptor (PXR) has been demonstrated suggesting regulation of SLCO1A2 expression may be achieved through PXR [20]. Supporting this, a 10-fold higher expression has been observed in BC *versus* healthy adjacent breast tissue, and SLCO1A2 expression is inducible in BC cell lines upon stimulation by the PXR activator, rifampicin, resulting in E-3-S uptake and proliferation [22].

### SLCO1B1 (OATP1B1) and SLCO1B3 (OATP1B3)

Physiologically, SLCO1B1 and SLCO1B3 are regarded as a largely liver-specific, localizing to the basolateral membrane of hepatocytes [5]. In cancer, they are expressed more widely and are typically overexpressed. SLCO1B1 overexpression has been demonstrated in colonic, ovarian, prostate and pancreatic cancers [16, 18, 23, 24]. SLCO1B3 is overexpressed in colonic, lung, breast, prostate, pancreatic, testicular and ovarian cancers [25–31]. SLCO1B1 expression in pancreatic cancer has been investigated as a potential target for selective toxicity to microcystin cyclopeptides [18]. SLCO1B1 is overexpressed in colon cancer and a significant relationship between SLCO1B1 expression levels and degree of differentiation in colon and liver cancer has been reported [29]. Higher mRNA levels of SLCO1B1 and SLCO1B3 in ovarian tissues and cell lines correlates with higher uptake rates of paclitaxel, suggesting a role for these transporters in the deposition of clinically relevant drugs [23].

SLCO1B3 overexpression in pancreatic adenocarcinoma compared with normal pancreas has been suggested as a potential marker of early-stage disease or as a target for drug treatments [32]. Likewise, its overexpression in colorectal cancer is suggested to confer a survival advantage by altering P53-dependent pathways [26]. A significant increase in SLCO1B3 expression has been shown to correlate with increasing Gleason score [29]. In contrast to their widespread overexpression in some cancers, several groups have shown that expression of both SLCO1B3 and SLCO1B1 is decreased in hepatocellular carcinoma at both the mRNA and protein levels [33–36]. This reduction may be attributed to de-differentiation in liver tumours and subsequent reduction in metabolic function [29].

While it has been presumed that the SLCO1B3 gene detected in cancers was identical to that seen in the healthy liver, a cancer-specific variant, OATP1B3 V1, is now known to arise due to alternative splicing. This variant differs from the wild-type (WT) protein as it lacks 28 amino acids in its N-terminus and is thought to reside predominantly in the cytoplasm rather than the plasma membrane like its WT counterpart and exhibits more restricted transport abilities of model substrates [31, 37].

There are conflicting reports of SLCO1B1 expression in BC cell lines and tissues. One study assessed SLCO1B1 expression in five paired samples of normal breast tissue and BC but found that SLCO1B1 expression was below the detection limit of qPCR [38]. Likewise, another study comparing expression in normal breast tissue and tumour samples found SLCO1B1 was not highly expressed in these tissues *via* qPCR, a similar finding to a study involving microarray analysis of human normal breast and cancer tissues [29, 39]. Conversely, assessment of mRNA expression in MCF10A, MCF-7, MDA435/LCC6, MDA-MB-468 and MDA-MB-231 cell lines showed expression to be highest in MDA-MD-231 cells and low, but detectable, in MDA435 and MCF10A cell lines. SLCO1B1 was also found to be expressed at the protein level with similar expression pattern, again with high expression in MDA-MB-231 cells [40].

As with SLCO1B1, there are contradictory reports of expression of SLCO1B3 in BC. In cell lines, SLCO1B3 was found to be expressed using qPCR in MDA-MB-231 and MCF10A but was not detected in the MCF-7 cell line [40]. Conversely, another study showed SLCO1B3 expression in the MCF-7 cell line *via* DNA microarray analysis [41]. However, in normal breast tissue samples and tumours, SLCO1B3 expression was detectable in two studies using both microarray analysis and qPCR [29, 39]. In contrast, another study examining SLCO1B3 expression in 102 breast carcinoma samples using IHC found SLCO1B3 expression in 50% of samples, and where immune reactivity was found this was inversely correlated to tumour size and was associated with a lower incidence of BC recurrence. The authors suggested that SLCO1B3 overexpression may be associated with a hormone-dependent growth mechanism and that expression of this transporter could be a potent prognostic factor in BC [28].

These conflicting reports may be due to a number of factors including; BC type, sample type and detection technique or could be due to expression of a cancer-specific variant gene, e.g. SLCO1B3 v1 found in colon and pancreatic cancers. Whether or not this is present in BC and involved in BC progression needs to be investigated.

Going forward, definitive analysis of the expression pattern of SLCO1B1 and SLCO1B3 in BC is important, since their substrate specificities suggest they may have a potential significance in different aspects of BC including cell growth (E-3-S and DHEA-S), chemotherapy (taxanes, methotrexate, rapamycin, methotrexate, paclitaxel, doxorubicin, doxertaxel, imatinib and SN-38), and also in diagnostic imaging, since it is recognised that gadolinium-based compounds such as gadolinium-ethoxybenzyl-diethylenetriamine pentaacetic acid (Gd-EOB-DTPA) are also substrates, and these are used in magnetic resonance imaging (MRI) to assess breast tumour enhancement [8, 14, 42–44].

Little is known about the role of SLCO1C1 in cancer/BC. It is predominantly expressed in the brain and testes and has been identified as a transporter of thyroid hormones [45]. No anticancer drugs have been identified as substrates to date; however, E-3-S and estradiol-17β-glucuronide are thought to be substrates [46]. SLCO1C1 is expressed in human osteosarcomas, metastasised renal cancers and aneurysmal bone cysts, though its function in these tissues has not been established [19].

SLOC2A1 is ubiquitously expressed and was originally identified as a prostaglandin transporter due to its high affinity for this substrate. Prostaglandins are thought to promote cancer progression by affecting cell proliferation, apoptosis, angiogenesis and immune response [5, 47]. Currently, no anticancer drugs are known to be transported by SLCO2A1; however, expression is known to be variable in cancer tissues. For example, SLCO1A2 expression was decreased in colon/rectum tumours as well as stomach, ovary, lung and kidney compared with normal tissues [47]. Conversely, increased expression has been shown in other cancers including breast, liver, and bone metastases from renal cancer [19, 48, 49].

SLCO2A1 expression is documented in several cancers including BC where, overall, it appears expression may be increased. A study comparing SLCO2A1 expression using qPCR in hormone-dependent BC cell lines (MCF-7 and ZR-75-1) to a breast epithelial cell line (MCF10A) found over a 3-fold increase in expression in the BC cell lines. The authors also assessed expression in 13 matched BC and non-malignant tissue samples *via* qPCR, and again, expression appeared to be increased in malignant tissue. Expression of SLCO2A1 *via* microarray analysis has also been demonstrated in normal breast tissue and breast tumour samples [39]. The apparent increased expression of this transporter in BC may be of significance since its main substrates are prostaglandins; increased intra-tumoural prostaglandins are considered indicators of poor prognosis in BC, as prostaglandins initiate signalling pathways associated with several hallmarks of cancer (angiogenesis, anti-apoptosis, proliferation, migration, invasion, immune evasion and epithelial mesenchymal transition and support of cancer stem cell-like phenotype). Another study investigated the expression of SLCO2A1 in different BC subtypes, and increased expression was seen in normal tissue and HER2-enriched or luminal A tumours than luminal B or basal. SLCO2A1 expression was also found to be significantly decreased in triple-negative breast tumours [50].

SLCO2B1 is another ubiquitously expressed transporter (Table 1), with highest expression reported in the liver [5]. Its substrate specificity is largely pH-dependent. At pH 7.4, its main substrates are steroid hormone conjugates; however, studies in the intestine show that increasing acidity increases transporter activity, with changes both in the substrate turnover and affinity [51]. Despite this broad and variable substrate specificity, to date, no cancer drugs are known to be substrates [8]. Nevertheless, SLCO2B1 expression has been shown in several cancers with some differences between malignant and non-malignant tissues. In bone, SLCO2B1 has been detected in both benign and malignant tumours, with significantly higher mRNA levels in aneurysmal bone cysts (benign) as compared with osteosarcomas (malignant) [19]. Likewise higher expression is seen in malignant *versus* non-malignant breast tissue, and expression increases with tumour grade [49, 52]. In the liver and pancreas, lower mRNA expression of SLCO2B1 in liver cancer was associated with decreasing differentiation and significantly lower expression of SLCO2B1 was seen in pancreatic cancer compared with normal pancreatic tissue. Conversely, expression was higher in thyroid cancer than normal tissue and expression increased with cancer stage [29].

SLCO2B1 has been identified in normal breast tissue and has been localised to the myoepithelium surrounding the ductal epithelial cells. This differs from invasive ductal carcinoma where it was found in the membrane of cytokeratin-positive epithelial cells [53]. However, the same study showed that in three BC cell lines (MCF-7, MDA-MB-231 and T47-D), SLCO2B1 remained under the detection limit *via* qPCR [53]. Likewise, several other studies have found SLCO2B1 expression to be undetectable or very low in cell lines *via* qPCR [40, 49, 54]. Protein expression also remained very low in cell lines [40]. SLCO2B1 expression at the transcriptional level has been detected in malignant and non-malignant tissue samples, with expression being higher in non-malignant tissues [49]. Conversely, another study analysing 120 tumour and 23 normal breast tissue samples using qPCR found higher expression in malignant *versus* non-malignant tissues, and that expression of SLCO2B1 increased with tumour grade, though neither of these findings reached statistical significance [52]. In agreement with previous studies, Kindla et al. detected SLCO2B1 in normal and malignant tissues however no statistical difference in expression was seen between the two groups. Their analysis using immunohistochemistry (IHC) and immunofluorescence (IF) showed SLCO2B1 expression was localised to the lobules/ducts and capillary epithelium in normal tissue but localisation in malignant tissue was mainly to the cytoplasm of malignant cells and plasma membrane of non-malignant cells [38]. SLCO2B1 expression in normal breast tissue and tumours is also supported by microarray analysis [39]. Among an array of substances transported by SLCO2B1, important substrates relevant to BC include E-3-S, DHEA-S and Prostaglandin E2 [14, 53, 55–57].

*SLCO3A1 (OATP3A1)* is also ubiquitously expressed showing highest expression in the testes, brain and heart (Table 1). This expression is the result of two splice variants; SLCO3A1_v1 and SLCO3A1_v2. While largely similar to the v2 variant, the v1 variant lacks 18aa in its COOH-terminal end. As a result, the v1 variant is widely expressed throughout bodily tissues whereas v2 expression is limited to testes and brain (Table 1) [58]. Substrate specificity remains largely similar between both variants and includes steroid hormones and conjugates, prostaglandins, and antibiotics [14]. SLCO3A1 has been detected in several cancers including breast, lung, colon, ovary and pancreas [57]. Additionally, higher SLCO3A1 expression has been found in several cancer types compared with normal or benign tissue, including, hormone-resistant metastases *versus* untreated prostate cancers, in primary and metastatic liver cancer *versus* normal liver, in aneurysmal bone cysts *versus* osteosarcomas and in BC cell lines *versus* non-malignant breast cell lines [19, 24, 40, 48].

In normal breast SLCO3A1 localises to the plasma membrane of lactiferous duct epithelial cells, but localisation alters in BC, shifting to the cytoplasm. This is not however associated with a change in expression level, as qPCR of matched normal and BC tissues showed no significant change in expression between the two groups [38]. Expression has been detected in breast cell lines *via* IHC (MCF-7, HBL-100 and BT-474) and qPCR (MCF-7, MCF-10A, MDA/LCC6–435, MDA-MB-468 and MDA-MB-231). Significantly higher expression was only seen between MDA435/LCC6 cells compared with MCF10A cells [40, 54]. In agreement with the molecular expression pattern, protein expression *via* western blot analysis showed significantly higher levels in MCF-7 and MDA435/LCC6 cells *versus* MCF-10A [40]. Wleck et al. also confirmed expression in MCF-7, ZR-75-1, MCF-10A and MDA-MB-231 cells, with significantly higher expression in the normal epithelial and hormone-independent cell lines compared with the hormone-dependent cell lines [49]. In tissues, expression was detected in both normal and malignant samples, with expression significantly higher in non-malignant tissue [49].

To establish if transporters play a role in E-3-S transport and proliferation in BC, uptake of E-3-S by T47-D and MCF-7 cells was measured and resulted in increased proliferation. SLCO3A1 and SLCO4A1 were identified as candidate transporters for this uptake *via* RT-PCR, and inhibition studies supported the notion that an OATP may be responsible [56, 59].

*SLCO4A1 (OATP4A1)* shares a similar ubiquitous expression pattern to SLCO3A1; likewise, it also transports E-3-S and prostaglandins [57]. SLCO4A1 expression has been shown in cancer cell lines derived from the breast, lung, colon, prostate, ovary and pancreas [57]. As for SLCO3A1, SLCO4A1 expression appears to be increased in aneurysmal bone cysts and liver cancers, BCs and prostate cancers [19, 24, 48, 49].

Studies show SLCO4A1 expression in several breast cell lines: MCF-10A, MCF-7, MDA/LCC6–435, MDA-MB-231, MDA-MB-468, ZR-75-1 and T47-D *via* a combination of molecular techniques (RT-PCR and qPCR) and protein analysis (Western blot) [40, 49, 53]. Expression was also seen in normal mammary gland tissue and matched normal, malignant and non-malignant breast tissue [49, 53]. Overall, these studies suggest that SLCO4A1 expression is higher in non-malignant *versus* malignant breast tissue, and higher in hormone-responsive *versus* hormone non-responsive BC [40, 49, 53].

Little is known about the role of SLCO4C1 in normal breast and BC. It was originally classified as kidney specific, however, while highest expression is seen in kidney moderate-to-low expression is also seen in breast, liver, lung and skin [39]. In the kidney, SLCO4C1 is localised to the basolateral membrane of proximal tubule cells where it facilitates excretion of exogenous drugs and endogenous compounds including methotrexate, thyroid hormones and E-3-S [5]. It remains poorly characterised in cancers in general, though microarray analysis has detected expression in renal, ovarian, leukaemia, lung, and glioblastoma cancer cell lines [60]. SLCO4C1 has also been detected in BC cell lines and tissues *via* qPCR [49]; expression was found in MCF-7, ZR-75-1 and MDA-MB-231 cells, with highest expression seen in MCF-7. Conversely, no expression was detected in the MCF-10A cells. In tissue specimens, 7 of 13 paired non-malignant and malignant samples showed higher expression in the non-malignant tissue [49]. Studies in rat have shown SLCO4C1 expression in breast epithelial cells significantly increases in lactating *versus* non-lactating tissue [61] indicating response to hormonal drive.

Both SLCO5A1 and SLCO6A1 remain poorly characterised in terms of transporter function and substrate specificity. SLCO5A1 may be expressed in the brain, prostate, skeletal muscle and thymus according to microarray analysis; however, this requires further verification [39]. SLCO6A1 was originally identified as testis specific; however, weak expression has been detected in normal spleen, brain and placenta [5]. Expression of both transporters has been detected in several cancers; for SLCO5A1, these include breast, bone, prostate, liver and small cell lung cancer [19, 38, 48, 62]. SLCO6A1 has been detected in lung cancer cell lines and tumours of the lung, bladder, oesophagus and brain [63, 64].

In terms of functionality, a study using SLCO5A1-HEK-293 transfected cells has shown increased resistance to Satraplatin, a potential chemotherapeutic agent for breast, prostate and lung cancer treatment [62].

Unsurprisingly, there is a paucity of information on the potential role of SLCO5A1 and SLCO6A1 in normal breast tissue and BC. SLCO5A1 expression has been shown *via* immunohistochemistry and immunofluorescence in the membrane of normal lactiferous ducts, but expression become less membrane-bound and more cytoplasmic in BC, showing a similar expression pattern to SLCO3A1 [38]. mRNA expression has been demonstrated in cell lines (MCF-7, ZR-75-1 and MCF-10A) and in normal and malignant tissue samples. In the cell lines, SLCO5A1 expression was 3-fold higher in MCF-7 cells than MCF-10A control cells. Conversely, in the tissue specimens, 8 of 13 normal tissue samples had higher SLCO5A1 expression than malignant tissues, however, the results were quite variable [49].

### OCTs and OCTNs

OCT1 is considered liver-specific, however weak expression has also been shown in other tissues such as heart, skeletal muscle, kidney and brain [65], and expression has been noted in several cancers [66]; (Table 2). Substrates include anticancer drugs such as imatinib and oxaliplatin [67]. Significant overexpression of OCT1 has been found in cell lines and samples from lymphoma patients, and this is thought to contribute to susceptibility to antineoplastic drugs [68]. Similarly, OCT1 is expressed in chronic myeloid leukaemia where it is associated with uptake of imatinib [69]. OCT1 has also been detected in healthy colonic tissues, colon cancer and polyps, where its overexpression is deemed a determinant of the anticancer effects of oxaliplatin [16, 70].

OAT2 is predominantly expressed in the kidney, however some expression is also seen in the liver, with comparatively low expression seen in many other normal tissues [71]. Notable substrates are platinum-based drugs (Table 2). OCT2 expression has been detected in glioma (SK-MG1) and colonic adenocarcinoma (Caco-2) cell lines [72] and its expression has been documented in 4 of 8 renal cancer cell lines, 4 of 6 ovarian cancer cell lines, 2 of 5 brain cancer cell lines and 1 of 8 colon cancer cell lines [73]. In tissue samples, OCT2 was present at very low levels in many tissues but absent in some cancer tissues including breast, colon, liver, lung, ovarian, prostate and thyroid [60].

OCT3 shows a different tissue specificity to both OCT1 and OCT2, and is more ubiquitously expressed, but shows stronger expression in the liver, placenta, kidney and skeletal muscle [74]. Investigations into OCT3 in cancer have mainly focused on it as a transporter of chemotherapeutic agents. Low OCT3 expression has been detected in human liver cell lines, normal healthy liver tissue and hepatocellular carcinoma (HCC), and it has been shown to be downregulated in HCC compared with healthy liver [75]. Conversely, it is highly expressed in colorectal cancer–derived cell lines compared with other OCTs and almost ten times more highly expressed in colorectal cancer cells than normal tissue. OCT3 highly expressing tissues are more sensitive to the cytotoxic effects of oxaliplatin, which has led to the suggestion that it may be used as a marker of efficacy of oxaliplatin treatment in colorectal cancer [76]. Likewise, OCT3 expression is seen in renal carcinoma cell lines and its expression has been linked to increased sensitivity to cytostatic drugs suggesting that the presence of OCT3 may be used to tailor therapies [77].

There are a few reports showing expression of OCT1–3 in breast tissues. In lactating and non-lactating mammary epithelial cells (MEC), OCT1 and OCT 3 but not OCT2 were detected, in lactating MEC, with OCT1 levels over 4-fold higher in lactating MEC *versus* non-lactating MEC [78]. In BC models, one study used qPCR to assess OCT1–3 expression in 9 cell lines; 4 luminal human BC (MCF-7, SK-BR-3, ZR-75-1 and BT-474) and 5 basal cancer cell lines (BT-20, MDA-MB-435S, MDA-MB-231, MDA-MB-468 and BT-549). In most of the cell lines, the levels of all three transporters were negligible; however, in MDA-MB-231, MDA-MB-468 and BT-549, their levels were relatively high [79]. Protein expression of OCT1 and OCT3 using Western blot analysis was also detected in MDA-MB-468, MDA-MB-435S and MDA-MB-232 cell lines. Using malignant breast tissue, non-malignant adjacent tissue and normal breast tissue, OCT3 showed the highest expression, followed by OCT1, with negligible OCT2 expression. Interestingly, OCT3 was lower in malignant compared with adjacent non-malignant tissue; however, this was not statistically significant [79].

OCTN1 is a transporter of zwitterions such as l-carnitine, which is required for beta-oxidation of fatty acids and ultimately energy production. This transporter shows ubiquitous expression but is more highly expressed in the kidney, trachea, and bone marrow (Table 2) [80]. To date, there is little evidence for a link between OCTN1 and cancer; however, expression has been documented in cancer cell lines including lung, colorectal, myelogenous leukaemia and HeLa [11, 80]. Expression of OCTN1 has however been linked to heightened sensitivity to mitoxantrone and doxorubicin [60]. Like the aforementioned cation transporters, OCTN1 transports oxaliplatin and its overexpression has been linked to drug accumulation and cytotoxic effects [81].

Like OCTN1, OCTN2 is a transporter of cations and is a sodium-dependent high-affinity transporter of carnitine. High OCTN2 expression is seen in various tissues including heart, placenta, skeletal muscle and kidney [82], as well as in several human cancer–derived cell lines including melanoma, lung, colorectal, chronic myeloid leukaemia and cervical carcinoma, showing a similar pattern of expression to OCTN1 [82]. The role of OCTN2 in cancer remains unclear, however one hypothesis is that its expression may be reduced in cancers bringing about a reduction in carnitine transport and in turn having knock-on effects for mitochondrial fatty acid ß-oxidation [83]. This is complementary to the Warburg effect which shows that cancer tumours favour a metabolic switch to energy production *via* glycolysis rather than β-oxidation regardless of the oxygen environment [84]. OCTN2 has also been identified as a transporter of both oxaliplatin and imatinib [85, 86].

OCTN2 has been localised to the luminal alveolar membrane of breast tissue [87]. It has also been identified as an oestrogen-dependent transporter associated with ER status in BC cells and tissues; its expression was confirmed in 15 BC cell lines using qPCR, with significant overexpression in ER-positive compared with ER-negative cell lines [88]. This was supported by cDNA microarray of BC cell lines and breast tumour tissue, and it was determined that OCTN2 acts as an oestrogen-activated intronic enhancer element that is crucial for carnitine homeostasis, lipid metabolism, and BC cell proliferation [88].

OCT6 is a carnitine transporter mainly expressed in the testis but also in the bone marrow, kidney and leukocytes [89]. RT-PCR has shown OCT6 expression in leukaemia cell lines and samples from leukaemia patients. Furthermore, uptake *via* OCT6 of doxorubicin, a commonly used treatment for acute myeloid leukaemia confers sensitivity in a leukaemia cell line [90, 91]. OCT6 is also highly expressed in liver- and colon-derived cancer tissues and cell lines, and is moderately expressed in cancer cell lines and tissues derived from prostate and uterus [91]. When SLC22A16 is highly expressed in testicular cancer there is increased sensitivity to treatment with bleomycin, a known substrate for this transporter [92].

### OATs

#### SLC22A6-A11 and SLC22A13

Of the 11 known OAT transporters, only SLC22A6 (OAT1), A7 (OAT2) A8 (OAT3), A11 (OAT4), A10 (OAT5), A9 (OAT7) and A13 (OAT10) are known to be expressed in humans [3]. OAT1–3 and OAT10 were originally identified as kidney specific while OAT5/7 and OAT4 were found to be expressed in the liver and placenta respectively. Although initially appearing to show tissue-specific expression, several of these have now been identified in multiple tissues (Table 2) [12]. Currently, the role they play in cancer is largely unknown. Some studies have suggested that they may transport anticancer drugs, for example 5-fluorouracil, a substrate for OAT2. High OAT2 expression is thought to be a predictor of good treatment response to 5-fluorouracil in colon cancer [93]. Similarly, methotrexate is transported by OAT1, OAT2 and OAT3 [94].

Expression in BC needs to be fully established; a study of 6 cancer cell lines revealed none expressed SLC22A6, A7 and A8 at the mRNA level, while analysis of human mammary mRNA samples revealed all were expressed [60].

*URAT1 (OAT4L)* is a kidney-specific transporter located in the apical luminal membrane of proximal tubule cells, where its main role is in the reabsorption of urate [95]. In humans, disease mutation in the URAT1 gene leads to hereditary renal hypouricemia; however, association of URAT1 with cancer remains un-investigated [96].

*SLC22A20 (OAT6)* was originally identified from the Ensembl mouse genome database, it is expressed in the nasal epithelium and, to a lesser extent, the testis [97]. It Is hypothesised to be involved in odorant detection due to its expression pattern and odorant substrates [98]. Additionally, OAT6 was demonstrated to transport E-3-S in *Xenopus* oocyte expression assays and Chinese hamster ovary cells transfected with mOAT6 [99]. To date, few connections have been made between SLC22A20 expression and cancer. However, analysis of RNAseq data showed expression in liver, kidney and thyroid carcinoma [74]. Further analysis *via* qPCR also showed expression of SLC22A20 in leukaemia and lung, kidney and liver carcinoma cell lines [100].

### Defining the role ole SLC transporters in breast Cancer

It is evident that there are differences in expression of solute carriers in different types of cancer and that this expression pattern can differ from that of healthy tissues. Major substrates for several SLC transporters include steroid hormones such as oestrogen conjugates and DHEA-S, as well as various anticancer drugs. As nearly two-thirds of newly diagnosed BCs are hormone dependent, it makes sense to look further at the role these transporters play in hormone-dependent BCs [101].

Estrogens are known promoters of BC cell proliferation. Free estrogens are lipophilic and so are able to freely diffuse across the plasma membrane. However, sulfo-conjugated steroid hormones (E-3-S) and the oestrogen precursor DHEA-S are hydrophilic and hold a net negative charge, thus are not freely diffusible. These hormones therefore require uptake transporters to enter cells. The SLCO/OATP subfamily has a potential role here, since estrogens are proven substrates for 7 of the 11 known human SLCO transporters: 1A2, 1B1, 1B3, 1C1, 2B1, 3A1 and 4A1 [102]. Although there is a decline in free estrogens in postmenopausal women the majority of hormone-dependent BCs occur in this group. This has been attributed to the biosynthesis of free estrogens from E-3-S and DHEA-S through the action of sulfatase and aromatase enzymes in BC cells that drive hormone-dependent cell proliferation [103]. In agreement with the role of estrogens as promoters of BC cell proliferation, it has been shown that oestrogen-dependent BC cell lines T47-D and MCF-7 proliferate on stimulation with E-3-S and that inhibition of E-3-S transporters results in the suppression of cell proliferation. The authors suggest several transporters may be responsible for this uptake of E-3-S and subsequent oestrogen-dependent proliferation of BC cells, but conclude that further investigation into these transporters is required [56, 59]. SLC transporters themselves may be regulated by hormonal stimulation, and this may occur through activation of PXR [20].

Current data pertaining to the clinical significance of SLC transporters in BC progression is mixed. Gene microarray results of 48,000 gene transcripts in 132 invasive breast carcinomas identified SLCO1B3 as a novel gene associated with the basal phenotype [104]. SLCO1B3 expression was associated with tumours of high histological grade, adverse survival and increased risk of early recurrence [104]. However, immunohistological analysis correlated with clinicopathological parameters in 102 breast carcinomas revealed SLCO1B3 expression is inversely correlated with tumour size and is associated with a decreased risk of recurrence [105]. In ER+ tumours, SLCO1B3 expression signified a good prognosis, and as oestrogen is a substrate for this transporters, it has been implicated in hormone-dependent growth mechanisms [105].

Thirty-one polymorphisms of the PXR, SLCO1A2, SLCO1B1 and SLCO1B3 transporters were analysed using MALDI-TOF MS and revealed that none of the 31 polymorphisms showed an association with breast cancer risk or tumour characteristics [106]. Polymorphisms were also investigated for SLCO1B1 (CYP2D6*10, A388G, T521C) in 296 hormone receptor-positive invasive breast tumours following adjuvant tamoxifen (TAM) therapy and revealed that there was a significant difference in overall survival between T521C and A388G but no difference in overall survival between CYP2D6*10 and A388G [107].

With respect to drug transport, SLCO1A2 and SLC22A16 expression has been investigated as a predictor of response to neoadjuvant chemotherapy. Immunohistochemical analysis was performed for 124 patients, pre- and post-anthracycline/taxane–based neoadjuvant chemotherapy. Analysis revealed that combined high OATP1A2/high OCT6 may be a potential predictor of pathological good/complete response to anthracycline/taxane-based chemotherapy in breast cancer, especially in triple-negative tumours [108].

Clearly, there is a great deal of information indicating the potential importance of SLCO transporters in BC development, as well as in its treatment, but more work needs to be done to develop a unifying picture of their precise roles.

Compared with the SLCO transporters, less is known about the role of SLC22 transporters in BC. There is clearly some evidence for expression of SLC22A1, SLC22A2 and SLC22A3 in breast tissue, their differential expression in normal *versus* malignant breast tissues, and their ability to transport certain anticancer drugs is certainly of potential significance [67]. Still, less is known about the expression and function of other SLC22 transporters. SLC22A6, SLC22A7, SLC22A8 and SLC22A11 are of potential interest because of their substrate specificities, which, like the SLCOs, include oestrogen conjugates and certain anticancer drugs [10, 13, 109]. Despite the apparent absence of SLC22A6, SLC22A7 and SLC22A8 in 6 BC cell lines, all were found in mRNA of human mammary tissue [60].

### Is there a role for SLC transporters in breast cancer metastasis?

The role of SLC transporters for all metastatic cancers remains largely unclear and primarily un-investigated. It has been reported that SLCO2A1, SLCO3A1, SLCO4A1 and SLCO5A1 are all expressed at both the mRNA and protein levels in both cancerous and non-cancerous liver tissue, including expression in liver metastasis [48]. In addition, in a study of primary small cell lung cancer (SCLC), using cells and tissue from both normal lung tissue and from lung biopsies and metastases of untreated SCLC patients, it was observed that while SLCO4A1 was the most widely expressed of 11 SLC transporters investigated, SLCO5A1 was the most highly expressed of the OATPs in a cell line derived from an SCLC patient liver metastasis (DMS153). Moreover, this patient had been treated with the chemotherapeutic reagents cytoxan and methotrexate [110]. This small emerging body on patterns of expression of SLC transporters associated with metastatic cancer supports their potential to contribute to development of drug-resistant metastatic disease and implies they could be novel therapeutic targets.

## Conclusion and next steps

There is enough preliminary evidence to suggest a potentially important role of solute transporters in BC. However, a comprehensive analysis of expression of all transport proteins in BC has yet to be carried out; once completed, it may be possible to paint a clearer picture of the roles of SLC family members, not only in the hormonal control of BC development but also as potential targets for hormonal and anticancer drug treatments. Functional experiments will subsequently help to establish definitive roles of SLC transporters in BC cells. Key to understanding their roles will be a better understanding of the interplay between SLC transporters, nuclear receptors, and ABC efflux transporters, which in turn will lead to the identification of targetable pathways to prevent progression to metastatic disease.

BC is a heterogeneous disease and expression profiling needs to take this into consideration. With a greater emphasis now on individualised therapies, this may be of significant importance in trying to reach that goal. In the future, genetic typing of transporter expression may even be used as a potential predictor of disease prognosis.

## Abbreviations

BC: Breast cancer
ABC: ATP-binding cassette
SLC: Solute carrier
OATP: Organic anion–transporting peptide
OAT: Organic anion transporter
OCT: Organic cation transporter
OCTN: Organic zwitterions/cation transporter
E-3-S: Estrone-3-sulfate
DHEA-S: Dehydroepiandrosterone sulfate
IHC: Immunohistochemistry
PXR: Pregnane X receptor
WT: Wild type
Gd-EOB-DTPA: Gadolinium-ethoxybenzyl diethylenetriamine pentaacetic acid
MRI: Magnetic resonance imaging
IF: Immunofluorescence
IHC: Immunohistochemistry
HCC: Hepatocellular carcinoma
